# Chicago Medical College

**Published:** 1866-03

**Authors:** 


### CHICAGO MEDICAL COLLEGE.

The exercises of the seventh annual commencement of this institution were held at the lecture-room of the college on the evening of March 1st.

The degree of Doctor of Medicine was conferred upon twenty-two candidates—the *ad eundem* degree upon four, and the honorary degree upon five.

After conferring the degrees, the president of the college, Prof. H. A. Johnson, addressed the class with a few very appropriate remarks.

The valedictory address was delivered by Prof. Byford. The subject of the address was, the "Philosophy of Trades and Professions," or the influences of the practice of medicine as compared with those of other occupations. It was received with great interest by the class, as well as by the rest of the audience. After the exercises, the class and their friends, with other members of the profession, were invited to a pleasant entertainment at the residence of Prof. Hollister.

---

Our readers could not fail to observe in our recent salutatory, the omission of the name of Prof. Allen, as one of the former editors of this Journal. In examining the earlier volumes of the Journal at the office of a friend, we carefully noted the names of those who had preceded us in the editorial chair. We cannot explain the manner in which the name of one who had during two years labored so faithfully for the Journal was omitted in our notice.

---

There has recently been on *exhibition* in this city a negro with an enormous enlargement of the scrotum, from a form of hypertrophy peculiar to one species of elephantiasis. The patient is twenty-two years of age, remarkably healthy in appearance, and of fine development. The disease of the scrotum commenced about eight years ago; it has not, apparently, progressed during the past twelve months. The tumor is said to be between 50 and 60 pounds, and to be 28 inches in length and 22 inches wide. Members of the professions interested in securing collections of photographs of medical and surgical specimens, can obtain a most excellent photograph of this patient and his tumor, by enclosing 30 cents to John Mountford, 272 South Clark street, Chicago.

---

Friends of Prof. Brainard will be interested to learn that letters have been received from him with intelligence that his health is much improved. On the 5th of February he was intending to leave Paris with his family to spend a couple of months in Italy. He states that his friends may expect him in Chicago as early as August or September next.

We have received the fee-bill of the Moultrie County Medical Society. The following abstract will give the general rate of charges: Prescription at office, with medicine, 75c. to $1.50. Visits in town, $1.50; to the country, $2 for first mile, and 75c. for each additional mile. For night visits, about a third more is added to the above. Obstetrical cases, $10; in the country, $10, with half mileage. Consultations, $8, with half mileage. Surgical operations, (with the exception of a few minor cases,) $10 to $500. The charge for tracheotomy is $100 to $300; for cataract, $100 to $500. In proportion to the expenses of living, we believe our friends in Moultrie co. are better paid for their professional services than most of our acquaintances in Chicago.

---

We are desirous of securing a complete series of the JOURNAL from its commencement in 1844 to 1858. It is stated that scarcely a single file of the JOURNAL is in existence. We have fortunately already collected several full and several incomplete volumes. We desire to procure the following numbers of the "Northwestern Medical and Surgical Journal:" for July, 1851; for May, July, November and December, 1854; for January, February and October, 1865; and for January, 1856. We shall be under obligations to any of our friends who can send us any of these numbers.

